# Digital Radiographic Study of Gonial angle in Forensic Odontology in a Tertiary Care Centre: A Descriptive Cross-sectional Study

**DOI:** 10.31729/jnma.5360

**Published:** 2021-04-30

**Authors:** Ritesh Srii, Sushmit Koju, Swagat Kumar Mahanta, Vinay Marla, Deepa Niroula, Chandan Upadhyaya, Prapulla Sridhara Murthy

**Affiliations:** 1 Department of Oral and Maxillofacial Pathology, Kathmandu University School of Medical Sciences, Dhulikhel, Kavre, Nepal; 2 Department of Oral and Maxillofacial Pathology, Kantipur Dental College, Basundhara, Kathmandu, Nepal; 3 Department of Community Dentistry, Kathmandu University School of Medical Sciences, Dhulikhel, Kavre, Nepal; 4 Department of Oral Pathology, Penang International Dental College, Malaysia; 5 Department of Oral Medicine and Radiology, Kathmandu University School of Medical Sciences, Dhulikhel, Kavre, Nepal; 6 Department of Oral and Maxillofacial Surgery, Kathmandu University School of Medical Sciences, Dhulikhel, Kavre, Nepal; 7 Elegant Dentistry, Breksville Road, Richfield, Oh-44286, United State of America

**Keywords:** *angle*, *forensic*, *lateral*, *malocclusion*, *panoramic radiograph*

## Abstract

**Introduction:**

Gonial angle measurements serve as an important parameter of the craniofacial complex and are usually studied on lateral cephalograms, but recently panoramic radiographs have also been used. The aim of this study is to measure the gonial angle using orthopantomogram and lateral cephalogram based on age, gender, ethnicity, and skeletal malocclusion in a tertiary care hospital.

**Methods:**

This is a descriptive cross-sectional study comprising 350 selected radiographs of apparently healthy individuals collected for a period of one year, from the Department of Oral Radiology of a tertiary care centre of Nepal. Gonial angle measurements were recorded from digital panoramic radiograph (both right and left side) and lateral cephalogram. Data was collected in terms of age, gender, ethnicity, and skeletal malocclusion and then statistically analysed using the Statistical Package for the Social Sciences version 20.

**Results:**

The mean gonial angle for panoramic right, left and lateral cephalogram was 122.490±7.570, 123.620±7.060, and 124.150±6.910, respectively. There was a decrease in the mean values of the gonial angle observed as age advances seen in all the radiographs. The gonial angle measured in Class III malocclusion was higher in all the radiographs, followed by Class II and Class I. Gonial angles obtained in females were higher than the males in all the radiographs. Finally, the Aryan population showed a higher gonial angle compared to the Mongolian population in all the radiographs.

**Conclusions:**

Panoramic radiograph (left side), could be considered as a reliable tool to measure the gonial angle.

## INTRODUCTION

Age estimation and identification of an individual by skeletal remains is an integral part of medicolegal proceedings where a forensic odontologist can play a crucial role.^1,2^ Mandibular angle or gonial angle is important for orthodontic treatment and age estimation in forensic medicine. It is a constantly changing anatomical landmark in the head which can be measured clinically or radiographically.^3,4^

Patient requiring an orthodontic treatment, and jaw surgeries are often subjected to extraoral radiographic investigations which includes panoramic adiography and lateral cephalogram. These radiographs can be used to evaluate the gonial angle easily by using modern digital software. As lateral cephalogram results in superimposing images, the reliability measurements of gonial angle becomes questionable.^5,6^

The aim of this study is to measure the gonial angle using orthopantomogram and lateral cephalogram based on age, gender, ethnicity, and skeletal malocclusion in a tertiary care hospital.

## METHODS

This is a descriptive cross-sectional study conducted on the digital radiographs obtained from the patients who were referred to the department of Oral Radiology, Kathmandu University School of Medical Sciences. The study was carried out from the period of January 2019-January 2020. Ethical clearance was obtained from the Institutional Review Committee. High-quality radiographs of apparently healthy individuals were included in the study. Radiographs of patients with a history of craniofacial syndromes/ malformation, fractures of the mandible, previous history of mandibular surgery, and post-orthodontic treatment were excluded from the study.

The sample size was calculated by using formula,

$$\begin{array}{ll} \text{n} & ={\text{Z}}^{\text{2}}\times \text{σ}/{\text{e}}^{\text{2}}\\ & ={\left(1.96\right)}^{2}\times {\left(0.5\right)}^{2}/{\left(0.6\right)}^{\text{2}}\\ & =267 \end{array}$$

where,

n = sample size
Z = 1.96 at 95% Confidence Interval
a = standard deviation e= margin of error, 6%

Taking non-response rate as 20%, the sample size of 320 was considered.

About 350 participants were enrolled by convienient sampling method.

**Figure 1.**
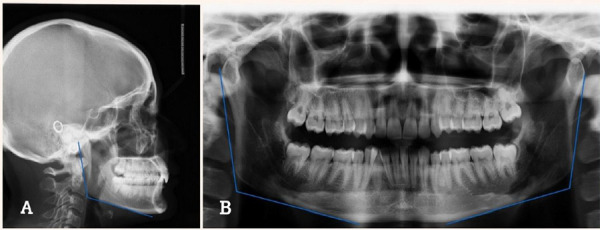
Gonial angle measurements on lateral cephalogram (A), and panoramic radiograph (B).

These radiographs were taken using Planmeca Promax machine using standard protocols as per specifications provided by the manufacturer. The radiographs were analysed using Planmeca Romexis software version 3.1.1.R. The gonial angle was measured at the point of intersection of mandibular plane and the ramal plane (Figure 1, A).

The mandibular plane was obtained by drawing a tangent in the lower border of the mandible. The gonial angle in panoramic radiograph as measured separately for the right and left side (Figure 1, B). Cephalometric analysis was done using Steiner's analysis, and the patients were categorized into three groups of skeletal malocclusions: Class I, Class II, and Class III. The selected patients aged between 7-52 years of age with a mean age of 21.539±5.626 years. They were categorized based on age into three different groups, group I: <18 years, group II: 18-25 years, group III: >25 years, followed by gender segregation comprising of 124 males and 226 females. Furthermore, the radiographs were segregated based upon ethnicity as group I-Mongolians (161) and group II-Aryan (189). The statistical analysis was done using statistical package for social sciences version 20.0 for Windows (SPSS Inc., Chicago, IL, USA). As the data recorded were quantitative, they were analysed using mean and standard deviation.

## RESULTS

The mean value of the gonial angle obtained from the panoramic right was 122.49, panoramic left 123.62, and lateral cephalogram 124.15 (Table 1).

**Table 1:** Descriptive statistics of gonial angle obtained from the panoramic radiograph (right, left), and lateral cephalogram (n = 350).

Variables	n	Mean	Standard Deviation
Panoramic radiograph (right)	350	122.49	7.57
Panoramic radiograph (left)	350	123.62	7.06
Lateral cephalogram	350	124.15	6.91

The mean age of the patients in the present study was 21.53±5.626 years. Measurements of gonial angle showed higher mean values in group I age patients in the radiographs taken (123.14±8.50, 124.42±7.28, 125.34±7.43), followed by group II patients (122.38±6.86, 123.46±6.86, 123.91±6.52) and group III patients (121.96±7.64, 122.97±7.16, 123.21±6.92) respectively (Table 2).

**Table 2:** Descriptive statistics of gonial angle taken from different digital radiographs showing age-related changes (n = 350).

Variables	Age group	n	Mean	Standard Deviation
Panoramic right	I	104	123.14	8.507
	II	155	122.38	6.866
	III	91	121.96	7.646
	Total	350	122.50	7.579
Panoramic left	I	104	124.42	7.282
	II	155	123.46	6.862
	III	91	122.97	7.162
	Total	350	123.62	7.069
Lateral Cephalogram	I	104	125.34	7.343
	II	155	123.91	6.527
	III	91	123.21	6.926
	**Total**	**350**	**124.15**	**6.911**

When malocclusion was considered, it was found that patients with skeletal Class II malocclusion were dominant in the present study representing about 204 (58.3%) followed by Class I; 104, (29.7%) and Class III; 42 (12%) respectively. Class III malocclusion patients showed higher mean gonial angle followed by Class II and Class I in all the radiographs (Table 3).

**Table 3:** Descriptive statistics of gonial angle taken from different digital radiographs showing changes related to skeletal malocclusion (n = 350).

Variables	Skeletal malocclusion (Class)	n	Mean	Standard deviation
Panoramic right	I	104	120.54	8.240
	II	204	123.04	6.737
	III	42	124.69	8.805
	Total	350	122.50	7.579
Panoramic left	I	104	122.18	7.808
	II	204	123.97	6.486
	III	42	125.50	7.379
	Total	350	123.62	7.069
Lateral cephalogram	I	104	122.46	7.064
	II	204	124.74	6.454
	III	42	125.48	8.010
	Total	350	124.15	6.911

Further segregation of data based on gender revealed, 124 (35.4%) male patients and 226 (64.6%) female patients. Female patients showed a higher mean value of gonial angle than the male patients in the radiographs taken. The least mean difference was seen in the lateral cephalogram followed by panoramic right and panoramic left (Table 4).

**Table 4:** Descriptive statistics of gonial angle taken from different digital radiographs showing gender- related changes (n = 350).

Variables	Gender	n	Mean	Standard deviation	Mean difference
Panoramic right	Male	124	121.38	8.584	-1.73
	Female	226	123.11	6.911	
Panoramic left	Male	124	122.47	7.523	-1.78
	Female	226	124.25	6.740	
Lateral Cephalogram	Male	124	123.63	7.774	-0.80
	Female	226	124.44	6.388	

Lastly when the gonial angles were segregated based on the ethnic groups it was seen that the mean gonial angles were higher in group II (Aryans) when compared to group I (Mongolians) in all the radiographs (Table 5).

**Table 5:** Descriptive statistics of gonial angle taken from different digital radiographs showing changes related to ethnic groups (n = 350).

Variables	Ethnicity	n	Mean	Standard deviation	Mean difference
Panoramic right	I	161	121.61	7.728	-1.64
	II	189	123.25	7.387	
Panoramic left	I	161	122.44	6.905	-2.18
	II	189	124.62	7.070	
Lateral Cephalogram	I	161	123.14	6.808	-1.86
	II	189	125.01	6.899	

## DISCUSSION

Variations in sexual dimorphism can be determined using skeletal remains. In cases of mass disaster, identification of an individual can be done only by means of skeletal remains. In such cases, mandible can play a vital role next to pelvis in age estimation as well as identification of an individual.^1,2^

In the facial skeleton, mandible is the only movable bone, and due to its inherent asymmetry, both sides of the mandible are not perfectly symmetrical.^7^ In mandible, gonial angle is the most used measurement in various aspects of dentistry. It is related to aesthetic, sleep disorders, orthodontics, risk of angle fracture, and in forensics.^8^

Extra-oral radiographs are an essential diagnostic aid used for orthognathic surgeries and in planning orthodontic treatments. Gonial angle is a frequently measured landmark in cephalometric analysis where super-imposition of the anatomical structures might result in variation in the actual value of the angle. Alternatively, panoramic radiograph can be a reliable source for measuring the gonial angle bilaterally.^9,10^

The values of the mean gonial angle obtained from the lateral cephalogram were higher followed by panoramic left and panoramic right radiographs. The mean difference observed between panoramic right, and left was (-1.13), and between panoramic right and lateral cephalogram was (-1.66). The least mean difference was observed between left panoramic radiograph and lateral cephalogram (-0.53). Based on these observations the determination of gonial angle from right panoramic radiograph remains questionable. On the other hand, the gonial angle measurements of the left side of the mandible could be considered to be reliable. However, studies involving an even larger sample size may be required to validate this. The variations in the gonial angle between right and left panoramic radiograph could be attributed due to the changes in masticatory forces.^11^

These results were similar to the study done by de Sousa, et al. in 2006^12^ where they observed a difference in the gonial angle between the right and left side of the panoramic radiograph, stating that the gonial angle on right side was significantly smaller than the left side. This difference could be due to more usage of right side due to chewing habits.^13^

Similar results were also found in a study done by Adil, et al. in 2015^14^ where they found differences in gonial angle between panoramic radiograph and lateral cephalogram.

Mattila, et al. in 1977^15^ conducted a study on dried skulls and concluded that orthopantomography can produce accurate measurements than the cephalogram. Gonial angle measurements obtained from panoramic radiograph are more reliable, as the cephalogram produces super imposing images which sometime becomes difficult to obtain accurate measurements. Also, the measurements can be obtained from both the sides of the panoramic radiograph which can be viewed more clearly.

The above statement has also been tested and proved by the study conducted by other authors.^16,17^

In the present study, as the mean difference observed between the panoramic radiograph left side and lateral cephalogram was minimal, it could be concluded that left side panoramic radiograph could be used to measure gonial angle provided the chewing habits of the subjects are recorded.

Apart from the determination of gonial angle panoramic radiograph can also be used to measure mandibular inclination. Both gonial angle and inclination of mandible are good indicators to evaluate the steepness and growth direction of the mandible. Panoramic radiograph is also commonly recommended by the dentists during routine examination and by the orthodontists to detect any vertical growth problems. This routine availability of such radiographs could be an asset of forensic importance.^18^

Fatahi and Babouei in 2007^19^ in their study on gonial angle measurements obtained using panoramic radiographs and lateral cephalograms and comparing them with measurements done on the dry skull manually, observed a high correlation between the panoramic radiograph and lateral cephalogram concluding that, panoramic radiograph was more reliable in determining the growth direction of mandible.

Further this statement was also validated and substantiated by authors including, Kurt, et al. (2008),^20^ Shahabi, et al. (2009),^6^ and Ongkosuwito, et al. (2009).^9^

The present study revealed a decrease in the gonial angle with increasing age. These results are in contrast with the study done by Al-Shamout, et al. (2012).^21^ which revealed an increase in the gonial angle as the age advances. Their study results revealed that the gonial angle and bigonial widths increased with age.

In our study, we observed that skeletal malocclusion Class II (58.3%) was more prevalent among the study samples followed by Class I (29.7%) and Class III (12%) respectively. It was also observed that, Class II patients had a gonial angle of 124.74°±6.45° measured in lateral cephalogram which contrasted with the study done by Shahabi, et al.^6^ in which they obtained a gonial angle of 125.00°±5.87° among the Class I malocclusion patients. Our study showed that patients with Class I malocclusion have less gonial angle than Class II and Class III patients as observed in all the radiographs.

The results of gender-wise comparison in the present study revealed a higher mean value of gonial angle in females than males in both the sides of the panoramic radiograph (mean difference right; -1.732, left; -1.784) and the least was found in the lateral cephalogram (mean difference -0.809). These results contrast with the study results done by Al-Shamout, et al (2012).^21^ in which males were found to have a higher gonial angle than the females. In few studies, it was observed that gonial angles in females were relatively higher (3°-5°) compared to the males.^5,22^

Raustia, et al. (1997)^23^ in their study done on edentulous patients (mean age-61 years) did not observe any differences in the gonial angle between the genders.

Gonial angles in humans vary from 100°-148°, which is found highest among the Caucasians and nearly as high in Negroes and the lowest angles are found among the Chinese, Australians, and American Indians.^25^ Rai, et al. (2007)^24^, and Thakur, et al. (2013)^25^, in their study found the mean gonial angle among North Indian population to be 114.3° and 119.04° respectively.^24,25^

Finally, results based on ethnic variation, revealed that group II (Aryans) had a higher mean value of gonial angle than the group I (Mongolians) in all the radiographic measurements taken. Further studies are required to characterize the differences in gonial angle to evaluate ethnic differences.

Since the study is based on the data of the Department of Oral Radiology, Dhulikhel Hospital, the findings cannot be generalised to the whole population.

## CONCLUSIONS

Gonial angle can be considered as an important parameter for evaluating age, gender, and ethnic variations. The easy availability of panoramic radiographs can be an important factor in considering it as a suitable technique for such an assessment. Our study revealed the gonial angle as measured on the left side of a panoramic radiograph could be a reliable source of measurement.
